# Permanent neonatal diabetes due to a novel insulin signal peptide mutation

**DOI:** 10.1111/pedi.12011

**Published:** 2013-01-28

**Authors:** Suhaimi Hussain, Johari Mohd Ali, Muhammad Yazid Jalaludin, Fatimah Harun

**Affiliations:** a Department of Paediatrics, School of Medical Sciences, Universiti Sains MalaysiaKelantan, Malaysia; b Department of Molecular Medicine, Faculty of Medicine, University of MalayaKuala Lumpur, Malaysia; c Department of Pediatrics, Faculty of Medicine, University of MalayaKuala Lumpur, Malaysia

**Keywords:** congenital absence of insulin-producing β-cells with diabetes mellitus, insulin, mutation, proinsulin, signal peptide

## Abstract

We report a rare case of permanent neonatal diabetes (PND) due to insulin (*INS*) gene mutation in a 51-month-old girl who presented with hyperglycemia in the neonatal period. Mutational analysis of *KCNJ11* and *INS* was performed and this detected a novel heterozygous c.38T>G (p.Leu13Arg) *INS* de novo mutation. The non-conservative change substitutes the highly conserved L^13^ residue within the hydrophobic core region of the preproinsulin signal peptide. Given the frequent tendency of heterozygous *INS* mutations to exhibit dominant negative disease pathogenesis, it is likely that the mutant preproinsulin perturbed the non-mutant counterpart progression and processing within the β-cells, and this resulted to a permanent form of congenital diabetes.

Neonatal diabetes (ND) is a form of monogenic diabetes that is usually defined as overt diabetes diagnosed during the first 6 months of life. A recent study quoted its minimal incidence as 1 in 90 000 1. ND cases include transient (TND) and permanent (PND) forms of diabetes, which display differing insulin dependency and molecular mechanism of disease pathogenesis 2, 3. Mutations in *KCNJ11*, *ABCC8*, and *INS* are among the major causes of PND 4, 5. *KCNJ11* and *ABCC8* encode the subunits of the ATP sensitive potassium channel (K_ATP_) of the pancreatic β-cells and activating mutations of these subunits could impair insulin secretions 6, 7. The insulin gene (*INS*) mutations are associated with PND and a spectrum of other clinical conditions such as type 1b diabetes, MODY, early onset type 2 diabetes, and TND 5, 8. PND cases could also arise from glucokinase (*GCK*) and insulin promoter factor-1 (*IPF-1*) mutations 9, 10. Syndromic cases associated with PND include rare mutations in *PTF1A*, *FOXP3*, *GLIS3*, *EIF2AK3*, *NEUROD1*, *RFX6*, *NEUROG3*, *GATA6*, *and SLC19A2* 11, 12.

## Case report

**Fig. 1:**
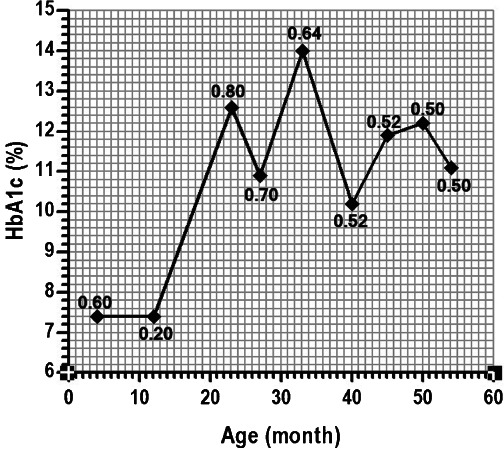
Proband hemoglobin A1c (HbA1c) levels were measured at selective visitations. In our practice, the variability shown is a typical feature for toddlers with early onset diabetes, which could have been caused by unpredictable eating behavior among subjects of such age. The numbers accompanying each point in the graph reflect the total dose of insulin (U/kg/d).

The proband is currently 51 months. She is the youngest of four siblings and there was no family history of diabetes. She was born at 36 wk via spontaneous vertex delivery with a birth weight of 1.7 kg (<third percentile), length 44 cm (<third percentile), and head circumference 32 cm (<third percentile). Her Apgar score was good, 9 at 1 min and 10 at 5 min. She was admitted to the neonatal ward in view of her low birth weight. From day 2 of life, she was noted to have persistently high blood sugar ranging from 15.0 to 30.0 mmol/L without ketonuria. There was no fever and her septic parameters were negative. Glutamic acid decarboxylase (GAD-65) antibody was negative and C-peptide was <165 pmol/L (normal range: 297.9–1324). Insulin was given by intravenous continuous infusion at 0.1 U/kg/h. The patient continued to have insulin dependency, but also had multiple episodes of hypoglycemia during intravenous insulin infusion. From day 10 of life, subcutaneous insulatard was started to replace intravenous insulin infusion. Achieving a good control of blood sugar was difficult as she was born small for the gestational age and at the same time she was very sensitive to insulin. To get a good match between her insulin and dietary intake, enteral feeding was optimized up to 200 mL/kg/d (130 kcal/kg/d). This regime helped to prevent hypoglycemia. The dose and frequency of insulatard was gradually increased to match her requirement. She was discharged on day 33 of life with insulatard, at a total daily dose of 0.6 U/kg/d. She was regularly followed up at the Pediatric clinic but it was difficult to have good glycemic control. Her hemoglobin A1c (HbA1c) ranged between 7.4 and 14.3%. The diabetic control deteriorated from the age of 2–3 yr with HbA1c reaching as high as 14.3% (Fig. 1). Insulin was then increased to 0.8 U/kg/d, with insulin aspart added in the insulin mixture. With that, she had a variable blood sugar pattern, with rapid drop of blood sugar, especially when insulin aspart is used. Her mother refused to include or use insulin aspart, as the child was very sensitive to it even at a small dosage of 0.5 U/kg/d. The blood sugar control was also difficult to achieve as the child had unpredictable eating habit and activity levels during her toddler's years. Recent evaluation at her current age showed that she had a normal glucagon level 63 pg/mL (normal range: 40–140), negative for anti-islet cell antibodies (ICA) and C-peptide <30 pmol/L (normal range: 297.9–1324). There was no hospital admission for severe hypoglycemia or diabetic ketoacidosis. Currently, her developmental milestones were appropriate for her age. She started to walk at the age of 12–13 months just like her other siblings, ride a tricycle at 3 yr, drawing a circle at 3 yr and speak 4–5 word sentences at about 4 yr. She has caught up in her physical growth with height 91.0 cm (third percentile) and weight 13.5 kg (third percentile).

## Mutational analysis of *KCNJ11* and *INS*

**Fig. 2:**
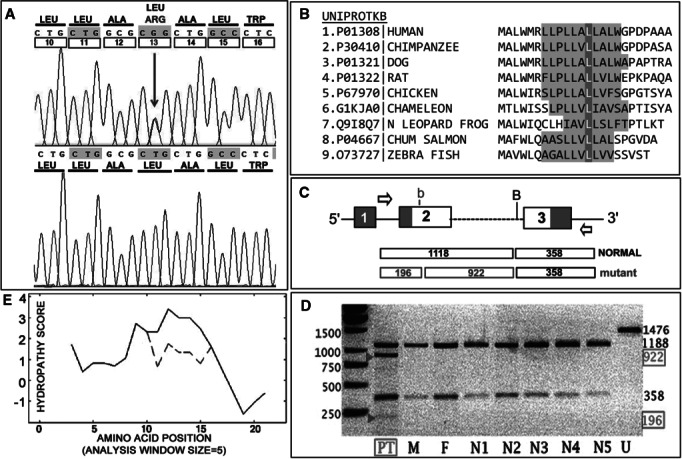
Analysis of *INS* p.L13R mutation. (A) Electropherogram for c.38T>G (p.L13R) heterozygous *INS* mutation (top) and the normal DNA sequence (bottom) for comparison. Boxed numbers indicate codon positions. (B) N-terminal Signal Peptide-alignment from representative chordates (mammals, bird, reptile, amphibian, and fish). Boxes indicate the archetypal N–H–C regions of an SP as described by von Heijne 20, where the hydrophobic core region (HCR) contains a minimum of seven hydrophobic residues interrupted by no more than one residue of either Gly, Pro, Ser, or Thr. The conserved L13 residue is highlighted. (C) *BstUI* map of 1476 bp PCR fragment amplified using flanking primers (arrows). The normal allele has only one *BstUI* site, whereas the p.L13R mutation created a novel *BstUI* site in exon 2. (D) PCR-RFLP analysis: the patient (PT) shows 922+196 bp *BstUI* RFLP associated with p.L13R mutation. M/F, maternal/paternal DNA; N1–N5, normal DNA; U, undigested control DNA. (E) Kyte–Doolittle plot 27 shows a significant drop in HCR hydrophobicity score (dashed line) upon p.L13R substitution.

Peripheral blood lymphocyte DNA was extracted using standard methods. Primers for all coding exons of *KCNJ11* and *INS* were designed to include ∼50–100 bp of intron–exon junctions. Purified polymerase chain reaction (PCR) products were directly sequenced. No sequence change was detected in *KCNJ11* but a heterozygous p.L13R *INS* mutation was found (Fig. 2A), that resides within the hydrophobic core region (HCR) of proinsulin (PI) signal peptide (SP). Direct DNA sequencing did not detect this mutation in the parental DNA. The mutation created a novel *BstUI* site. A 1476-bp fragment was generated using the primer pair GATTCCAGGGTGGCTGGAC and CCTGGCCGGCGTTGGCACC, which flanked the mutation site and was then subjected to *BstUI* digestion (Fig 2C, D). The variant was not detected in dbSNP (Build 137) and 162 control chromosomes derived from apparently healthy volunteers. The parent–proband biological relationship was positively confirmed (probability >99.99%) and this was deduced through DNA profiling using the following 15 STR loci: D8S1179, D21S11, D7S820, CSF1PO, D3S1358, TH01, D13S317, D16S539, D2S1338, D19S433, vWA, TPOX, D18S51, D5S818, and FGA.

## Discussion

PND due to *INS* mutations was first reported in 2007 4. Most cases are due to *de novo* mutations, although some show autosomal dominant inheritance. Heterozygous *INS* mutations are quite common as have been reported by various groups 8, and they can be dominantly acting and cause among others non-mutant PI misfolding, endoplasmic reticulum (ER)-retention/stress, abnormal insulin secretion, and β-cell apoptosis 13–15.

The proband's undetectable C-peptide, hyperglycemia, and growth retardation features which occurred very early in life suggested severe insulin deficiency, which have already begun *in utero*. The absence of GAD and ICA antibodies suggested a non-autoimmune disorder. The proband has thus far showed an appropriate developmental age and has caught up in her physical growth, without evidence of neuropsychological or neuromotor dysfunctions, features that have been observed in some PND cases with K_ATP_ channel mutations 16.

Our proband represents the first PND case with heterozygous *INS* mutation that occurred within the HCR of PI SP and resulted in an early onset of diabetes. Other SP-residing *INS* pathogenic mutations include heterozygous p.R6C/H and p.A24D 4, 5, 14, but these mutations seem to show lesser disease severity compared to p.L13R. The p.R6C/H carriers had milder clinical course, normal birth weight, and a later age of diabetes onset, consequently them being classified as MODY cases. The p.R6H substitution is a conservative change and only caused mild ER-stress without significant disruption of the mutant PI release in HEK293 cells, while functional analysis using MIN6 β-cells indicated p.R6C/H substitution did not impair mutant-PI vesicular targeting 14. Patients with p.A24D mutation showed variable disease presentation and a later age-at-diagnosis ranging from 4 wk to 7 yr 5. Functional assay using MIN6 β-cells indicated p.A24D mutant is retained in the ER and causes ER-stress, but it did not significantly affect wild type insulin secretion 15.

**Fig. 3:**
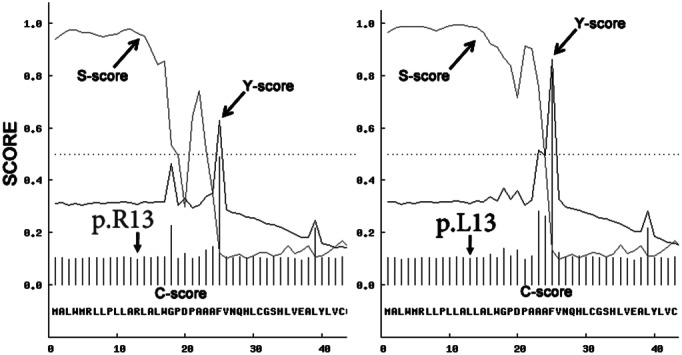
Signal P 4.0 analysis output for p.L13R *INS* mutation. INS-SP covers residues 1–24. The presence of a signal peptide is predicted by scoring every residue (‘S-score’) for the stretch of amino acids analyzed. The predicted score for signal peptidase cleavage site is indicated by the C-score. Y-score is a composite score for C- and S-scores, to improve cleavage site prediction, where its value would increase when the slope of the S-score is steep and a significant C-score is found. (www.cbs.dtu.dk/services/SignalP-4.0/output.php).

The p.L13R substitution is likely to compromise insulin SP functions. SignalP 4.0 analysis indicated the authentic cleavage site score for p.L13R is reduced from ∼0.9 to ∼0.5 17 (Fig. 3). Such indication seems to agree that HCR modifications can affect signal peptidase cleavage activity as previously reported 18. PolyPhen-2 and SIFT analysis 19 predicted p.L13R mutation as likely to be pathogenic. Such mutation is a non-conservative change, substituting a neutral-hydrophobic residue into a polar or charged-hydrophilic amino acid. This introduced an abnormality as polar and charged amino acids are virtually absent in the HCR 20. An introduction of a charged residue in the HCR is functionally disruptive and not tolerated 21. An intact HCR is crucial for the SP interaction with signal recognition particle and the ER-membrane, so that the co-translational translocation process of insulin synthesis could occur correctly 22. Other reports have shown that similar HCR disruptions were functionally detrimental. The same missense substitution (L→R) in the HCR was able to block the periplasmic export of the affected secretory proteins in the prokaryotic system 23, 24. A heterozygous p.L25R *COL5A1* mutation within the HCR resulted to the classic Ehlers–Danlos syndrome 25. A homozygous substitution p.L15R in the bilirubin UDP-glucoronyltransferase (B-UGT) HCR causes the development of type II Crigler–Najjar disease 26. The mutant B-UGT translation and processing were markedly impaired in the presence of the microsomal fraction, indicating a detrimental interaction of the mutant B-UGT with the ER-machinery. The mutation may have impaired the enzyme ER-translocation, causing defective processing and its eventual degradation. Although B-UGT is membrane bound and insulin is instead a secretory protein, their common ER-targeting and processing requirement may be similarly impacted by the similar HCR disruption, albeit a dominant acting disease pathogenesis is expected for *INS* p.L13R mutation. This is based on the typical behavior of numerous heterozygous *INS* mutations reported to date 8, 13, 16. Such scenario is conceivable as our proband had an undetectable C-peptide, which strongly suggests that, the non-mutant PI processing or secretion is impaired through the usual dominant negative disease mechanism.

The case presented here suggests that a heterozygous *INS* mutation which disrupts the HCR can cause severe insulin deficiency with very early onset of diabetes and *in utero* growth retardation. A detailed functional analysis is warranted, as it could shed more insights on how such HCR disruption negatively affects insulin trafficking, maturation, and secretion in β-cells and eventually leading to a severe disease development. The identification of specific genetic cause contributing to PND guides us in the treatment decision, as patients with *INS* mutations may experience severe β-cells dysfunction that requires supplemental insulin for the rest of their life.
